# Future Prospects of Immunotherapy in Non-Small-Cell Lung Cancer Patients: Is There Hope in Other Immune Checkpoints Targeting Molecules?

**DOI:** 10.3390/ijms23063087

**Published:** 2022-03-13

**Authors:** Natalia Krzyżanowska, Kamila Wojas-Krawczyk, Janusz Milanowski, Paweł Krawczyk

**Affiliations:** Department of Pneumonology, Oncology and Allergology, Medical University of Lublin, 20-954 Lublin, Poland; kamilawojas@wp.pl (K.W.-K.); janusz.milanowski@umlub.pl (J.M.); krapa@poczta.onet.pl (P.K.)

**Keywords:** non-small cell lung cancer, immunotherapy, immune checkpoints, immune checkpoint inhibitors, immune checkpoint agonists

## Abstract

Currently, one of the leading treatments for non-small-cell lung cancer is immunotherapy involving immune checkpoint inhibitors. These monoclonal antibodies restore the anti-tumour immune response altered by negative immune checkpoint interactions. The most commonly used immunotherapeutics in monotherapy are anti-PD-1 and anti-PD-L1 antibodies. The effectiveness of both groups of antibodies has been proven in many clinical trials, which have translated into positive immunotherapeutic registrations for cancer patients worldwide. These antibodies are generally well tolerated, and certain patients achieve durable responses. However, given the resistance of some patients to this form of therapy, along with its other drawbacks, such as adverse events, alternatives are constantly being sought. Specifically, new drugs targeting already known molecules are being tested, and new potential targets are being explored. The aim of this paper is to provide an overview of the latest developments in this area.

## 1. Introduction

At present, clinicians have a variety of options for treating advanced non-small-cell lung cancer (NSCLC) patients. Molecularly targeted therapies are one of these options. In these therapies, patients are screened for mutations that predispose them to respond to personalised treatment. The studied genes include *EGFR* (epidermal growth factor receptor), *ALK* (anaplastic lymphoma kinase), *ROS1* (ROS proto-oncogene 1), *BRAF* (proto-oncogene B-Raf), *MET* (mesenchymal-epithelial transition factor), *RET* (RET proto-oncogene), *NTRK 1–3* (neurotrophic tyrosine kinase 1-3), and *KRAS* (Kirsten rat sarcoma virus) [1]. In non-molecularly-predisposed NSCLC patients, treatment relies on immunotherapy, which is based on immune checkpoint inhibitors (ICIs), which are monoclonal antibodies that work against negative co-stimulatory molecules [2,3]. Physiologically, inhibitory immune checkpoint pathways help to maintain self-tolerance and to control the anti-microbial immune response. However, negative immune checkpoint molecules can be expressed in the tumour microenvironment (TME) and may therefore be involved in the evasion of host immune surveillance; accordingly, a blockade of those pathways enables anti-tumour immune response restoration [4].

Currently, in the immunotherapy of cancer, the most common targets within negative immune checkpoints are PD-1 (programmed death 1), its ligand PD-L1 (programmed death ligand 1), and CTLA-4 (cytotoxic T lymphocyte antigen 4) molecule when combination therapy is considered [3]. At present, the only validated biomarkers that qualify for ICI treatment in cancer patients are the percentage of tumour cells and/or immune cells with PD-L1 expression, as well as high microsatellite instability (MSI). PD-L1 expression on tumour cells, despite not being an ideal one, is the only predictive marker for immunotherapy in NSCLC patients so far proven in prospective clinical trials [5,6]. Of all the methods researched thus far, ICIs are the most efficient type of immunotherapy in advanced NSCLC. Nevertheless, those ICIs that are currently used are not always effective, and durable response is observed in a minority of patients. Unfortunately, in patients with PD-L1 expression on tumour cells, a lack of immunotherapy response could be observed, and this treatment method may also be effective in patients without PD-L1 expression [5].

Three possible situations of systemic progression are described in the clinic: early, intermediate, and late progression, and different immunological mechanisms are thought to be responsible for them (Table 1) [7]. It should be kept in mind that the mechanism of the anti-tumour immune response is extremely complex, involves multiple stages, and depends on many factors. While progression is often linked to neoantigen depletion or defects in IFN-γ signalling, some authors have described the upregulated expression of other negative immune checkpoints on T cells at the time of acquired resistance. These include TIM-3 (T-cell immunoglobulin and mucin domain 3), TIGIT (T-cell immunoreceptor with Ig and ITIM domain), and LAG-3 (lymphocyte-activation gene 3) molecules [8]. However, it has also been postulated that the stimulation of positive immune checkpoints, such as OX40 and CD137, can reverse ICI resistance. This has shifted researchers’ attention to the therapeutic potential of the blockade or stimulation of immunological checkpoints other than PD-1/PD-L1 and CTLA-4. These drugs are also not free of side effects, which are also known as immune-related adverse events (irAEs). As peripheral tolerance pathways are blocked mostly by anti-CTLA-4 or anti-PD-1 antibodies, these side effects have an autoimmune character and can affect most organs [9]. Therefore, searching for new antibodies and therapeutic targets is still the main subject of research for many oncologists.

**Figure 1:**
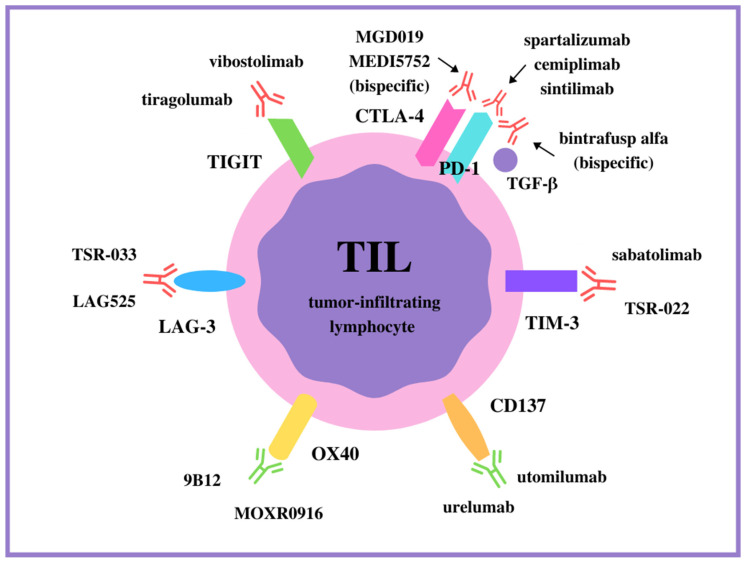
The monoclonal antibodies targeting positive (in green) and negative (in red) immune checkpoints on T cells.

The aim of this paper is to point out novel approaches, immunological targets, and hopes in NSCLC immunotherapy. In addition to combination therapy, targeting checkpoints other than the PD-1 and CTLA-4 pathways are presently being investigated. The new molecules recently considered for immunotherapy targets include both activatory and inhibitory checkpoints, and this review will focus on them. The results of preclinical studies on these molecules are so encouraging that they have led to the continued use of new ICIs in further phases of clinical trials. All the receptors described and the antibodies targeting them are symbolically shown in Figure 1.

## 2. Pathway Inhibitors Other Than PD-1/PD-L1: Discovering New Immune Checkpoint Inhibitors

The 2018 Nobel Prize in Physiology or Medicine awarded to Professors Tasuko Honjo (for his work on PD-1) and James Allison (for his work on CTLA-4) was undeniable proof of the enormous contribution of the discovery of these two negative control points to modern cancer treatment. However, it should be remembered that the list of molecules against which we can direct antibodies to block their functions is still incomplete. Cancer cells can successfully evade anti-PD-1 or anti-PD-L1 immunotherapy by expressing other negative molecules and extinguishing the activity of the immune system [8]. Other negative checkpoints are discussed below, along with the results of the clinical trials involving them.

### 2.1. TIGIT

The T-cell immunoreceptor with the Ig and ITIM domain (TIGIT, also known as the Washington University cell adhesion molecule [WUCAM], V-set and transmembrane domain-containing protein 3 [Vstm3], and V-set and immunoglobulin domain-containing protein 9 [VSIG9]) belongs to the poliovirus receptor-like (PVR-like) family [10]. It is expressed by lymphocytes, including CD4^+^ lymphocytes, CD8^+^ lymphocytes, Tregs (T regulatory cells), memory T cells, and natural-killer (NK) cells [10,11,12,13]. TIGIT is an inhibitory receptor and has three ligands: CD155, CD112, and CD113, which are expressed mainly by antigen-presenting cells (APCs), haematopoietic cells (CD112-positive), and non-haematopoietic tissues (CD155-positive, CD112-positive, and CD113-positive) [14,15,16].

While the exact effects of TIGIT interaction with its ligands are still being studied, it has been described as acting through several mechanisms. First, it is thought to transmit inhibitory signals to T cells and NK cells directly through its cytoplasmic tail via an intrinsic mechanism [12]. Second, in the cell-extrinsic mechanism, action on the TIGIT-CD155 axis increases the secretion of anti-inflammatory cytokines and decreases the secretion of proinflammatory cytokines by dendritic cells (DCs); these processes lead to impaired T-cell activation [10]. Another study revealed that TIGIT causes polarisation of CD155-expressing type 1 proinflammatory macrophages into IL-10-secreting type 2 macrophages (which are tumour-promoting) in mice [17]. In another mechanism, TIGIT may outcompete DNAX accessory molecule-1 (DNAM-1, CD226) from interacting with CD155 on macrophages, as these two molecules share CD155 as a receptor, and TIGIT binds to CD155 with higher affinity than DNAM-1 (expressed mainly by T cells, NK cells, and monocytes) [10]. Furthermore, TIGIT may disturb cis-homodimerisation on the cell surface and thereby prevent DNAM-1 interaction with CD155b [18]. Considering the nature of the DNAM-1 molecule, which co-stimulates cytotoxic T cells (Tc), the expected effect of its blockade is the inhibition of these cells’ function. It is worth adding that TIGIT-positive Tregs are described as cells with an activated phenotype that could suppress T cells [19].

TIGIT is found to be expressed by tumour-infiltrating lymphocytes (TILs) in NSCLC and ‘distant lung-associated lymphocytes’ (DLALs), but also by peripheral blood mononuclear cells (PBMCs) and, less significantly, tumour-free lung lymphocytes (TFLLs) [20,21]. Johnston et al. reported TIGIT overexpression by NSCLC-infiltrating CD8-positive T cells and its positive correlation with PD-1 expression [18]. Moreover, researchers have shown that there is synergistic action of PD-1 and TIGIT in tumour escape from immunological surveillance in a mouse model. Furthermore, the simultaneous blockade of PD-L1 and TIGIT was found to be much more effective than a single anti-TIGIT or anti-PD-L1 antibody [18]. Another study reported that TIGIT-positive NK cells infiltrating mouse subcutaneous tumours or human endometrial cancers were found to co-express other inhibitory receptors, such as LAG-3 and TIM-3, and their functions were altered [22]. Importantly, CD155 and CD112 are expressed by lung cancer cells or other cells in TME (e.g., tumour-associated macrophages [TAMs] with CD112 expression) [23,24]. Given the wide range of actions and presence in TME, TIGIT appears to be a promising therapeutic target in cancer treatment.

There have already been some clinical trials with anti-TIGIT monoclonal antibodies (mAbs). Vibostolimab, an anti-TIGIT antibody, was tested in patients with advanced NSCLC naive to prior anti-PD-1/PD-L1 therapy in a dose-finding study. In this case, vibostolimab was administered with pembrolizumab (anti-PD-1 mAb) and was well tolerated. The overall response rate (ORR) and progression-free survival (PFS) reached promising values, especially in patients with a tumour proportion score (TPS) of PD-L1 expression ≥1% (i.e., more than 1% of tumour cells with PD-L1 expression). The ORR was 46% (95% CI: 19–75) in the TPS ≥1% group and 25% (95% CI: 5–57) in TPS <1% group. The median PFS reached 8.4 months (95% CI: 3.9–10.2) in the TPS ≥1% group and 4.1 months (95% CI: 1.9-NR) in TPS <1% group. The median duration of response (DoR) for all patients was not reached (ranging from 4 to up to 17 months). Adverse events typical of immunotherapy occurred in 34 patients (83%) [25]. There are two other clinical trials with vibostolimab worth observing that are recruiting NSCLC patients. The NCT04738487 study is intended to verify the efficacy of the coformulation of pembrolizumab/vibostolimab in comparison to pembrolizumab monotherapy for PD-L1-positive metastatic NSCLC patients [26]. The NCT04725188 trial was designed to compare pembrolizumab/vibostolimab coformulation or pembrolizumab/vibostolimab coformulation plus docetaxel for metastatic NSCLC patients with progressive disease after platinum doublet chemotherapy and immunotherapy [27].

Another anti-TIGIT mAb, tiragolumab, was tested in a phase 2 trial in combination with atezolizumab in chemotherapy-naive patients with locally advanced PD-L1-selected NSCLC. At the beginning of 2021, tiragolumab was granted breakthrough therapy designation by the Food and Drug Administration (FDA) for use in combination with atezolizumab (anti-PD-L1 mAb) in the first-line treatment of metastatic NSCLC patients whose tumours have high PD-L1 expression but no *EGFR* mutations or *ALK* rearrangements. The most updated and critical results of the CITYSCAPE trial were presented at the ESMO Immuno-Oncology Congress in 2021. The ORR and median PFS in the tiragolumab plus atezolizumab group reached 38.8% (95% CI: 26.4–51.2) and 5.6 months (95% CI: 4.2–10.4), respectively. In contrast, in the placebo plus atezolizumab group, the ORR was 20.6% (95% CI: 10.2–30.9) and the median PFS was 3.9 months (95% CI: 2.7–4.5). In the subset analysis, significantly prolonged PFS was observed in the group with PD-L1 expression ≥ 50%, reaching 16.6 months (95% CI: 5.5–22.3), and the ORR reached 69.0% (95% CI: 50.4–87.5). Safety profiles were similar (Table 2) [28]. The CITYSCAPE trial is the first randomised trial of an anti-TIGIT therapy and provides evidence that targeting both TIGIT and PD-L1 molecules (tiragolumab plus atezolizumab) may improve anti-tumour activity by amplifying the immune response in advanced NSCLC patients [28].

### 2.2. TIM-3

TIM-3 (CD366) is an immunoglobulin and mucin domain-containing membrane protein and a member of the TIM family originally found to be expressed on terminally differentiated IFN-γ-producing Th1 (T helper cells; not on Th2) and Tc1 (T cytotoxic cells) murine cells [29]. It was then confirmed in human cells that Th17 cells are also regulated by this receptor [30]. TIM-3 is constitutively expressed by NK cells and DCs [31,32]. Gautron et al. demonstrated that TIM-3 can enhance the suppressor function of FoxP3^+^ Tregs with TIM-3 expression towards Th1 and Th17 responses [33]. This molecule has several ligands, which are shown in Table 3, together with the effects of their interaction with TIM-3 in the context of cancer (Table 3). The role of the TIM-3/phosphatidylserine (phosphatidylserine, PtdSer) axis in malignancies is unclear. It was shown in a murine model that TIM-3/PtdSer interaction may play a role in the clearance of apoptotic cells [34]. It has also been established to be present on human NSCLC cells and in vascular endothelial cell membranes at the tumour site [35,36].

It has been demonstrated that TIM-3, like TIGIT, could promote type 2 macrophage polarisation [41]. Also, TIM-3 can be present on an exhausted subset of CD8-positive TILs and CD4-positive Tregs in TME [42,43]. TIM-3 was also discovered in tumour-infiltrating DCs and found to suppress anti-tumour immunity [32]. As for NSCLC, TIM-3, along with LAG-3 and PD-1, was found in TILs and connected with their activation, as well as with a pro-apoptotic T-cell phenotype [44].

Clinical studies on anti-TIM-3 agents are still in their early stages. As mentioned above, resistance to immunotherapy has been linked by some researchers to the upregulation of TIM-3 molecule expression [45]. Considering this, many trials have focused on the combinatorial approach. Patients with different solid tumours, including NSCLC (without prior anti-PD-1/PD-L1 therapy), were enrolled in a trial with sabatolimab (MBG453), an anti-TIM-3 mAb, alone and in combination with spartalizumab (anti-PD-1 antibody, PDR001). In phase 1/1b, no responses were observed in the monotherapy arms using both of these immunotherapeutics [46]. Acceptable tolerability but limited efficacy was observed in the partial results for combination therapy. In phase 2, 41.2% (7/17) patients had stable disease (SD) per RECIST 1.1 (response evaluation criteria in solid tumours), 3/17 patients had durable clinical benefit (complete or partial response [CR or PR] or SD for ≥6 months), 3/17 obtained stable disease for <6 months or progressive disease (PD), and 1 patient was defined with unknown clinical benefit [47]. The AMBER trial (NCT02817633) aimed at testing different combinations of the anti-TIM-3 antibody (TSR-022) with the anti-LAG-3 antibody (TSR-033) and anti-PD-1 antibody (TSR-042) and different chemotherapy regimens in patients with solid tumours. One cohort recruited TIM-3-selected NSCLC patients (Table 2) [48].

### 2.3. LAG-3

LAG-3 (also called CD223) is a cell surface molecule and another inhibitory receptor. As a homolog to CD4, it binds to major histocompatibility complex (MHC) class II molecules, as well as liver sinusoidal endothelial cell lectin (LSECtin) and the galectin-3 molecule, negatively regulating T-cell functions [49,50,51]. It is expressed on activated CD4-positive and activated CD8-positive T cells, NK cells, B cells, and plasmacytoid dendritic cells [52,53,54,55]. Although LAG-3 mechanisms of action are still being investigated, we can easily summarise this mechanism by stating that LAG-3 can inhibit T-cell activation. It has been postulated that a unique ‘KIEELE’ motif (an amino acid sequence in the LAG-3 cytoplasmic tail) is essential for signal transduction of the LAG-3 molecule [56]. LAG-3 is involved in decreasing cytokine and granzyme production and the proliferation of T cells [57]. It also mediates Tregs’ regulatory function and is required for the full suppressive activity of natural and induced Tregs [58]. The LAG-3 ligand, galectin-3, was found to negatively regulate CD4^+^ and CD8^+^ T cells in murine models [51,59].

Kouo et al. observed suppression of CD8-positive T-cell function by LAG-3/galectin-3 axis in TME and inhibiting expansion of plasmacytoid DCs [51]. Importantly, expression of the galectin-3 gene was found in human NSCLC cell lines [60]. LAG-3 also binds to LSECtin expressed by DCs. Xu et al. observed that if LSECtin is present in melanoma cells, it inhibits the proliferation of tumour-specific effector T cells and thus enables immune escape [50]. Furthermore, LAG-3 marks Tregs releasing immunosuppressive cytokines, such as IL-10 and TGF-β1, with a more suppressive phenotype than LAG-3-negative cells from the tumour sites of cancer patients [61]. In a murine model, LAG-3 blockade led to augmentation of the amount and effector function in CD8^+^ T cells [62]. High expression of this receptor was found on TILs in the TME of some patients with NSCLC as well, and was positively correlated with PD-1 expression and related to poor prognosis [63]. Woo et al. indicated that, like TIGIT, LAG-3 may act synergistically with PD-1 to promote tumoural immune escape, and that combination anti-LAG-3/anti-PD-1 immunotherapy appears to be a legitimate strategy for cancer immunotherapy [64]. In NSCLC patients treated with PD-1/PD-L1 blockers, high LAG-3 expression was associated with a shorter PFS (*p* = 0.0314) [44].

Antibodies targeting LAG-3 are currently under investigation in different clinical trials. Ieramilimab (LAG525), a humanised anti-LAG-3 mAb alone or in combination with, remarkably, spartalizumab (PDR001), an experimental anti-PD-1 mAb, was tested in a phase 1/2 study (NCT02460224). As primary results showed, among 121 patients with advanced solid malignancies treated with LAG525 and spartalizumab combined therapy, 11 partial responses and 1 complete response assessed by RECIST were observed. Interestingly, in triple-negative breast cancer biopsies, investigators observed a trend in the conversion of immune-cold to immune-activated biomarker profiles (Table 2) [65]. ‘Hot’ tumours are defined as exhibiting heavy infiltration of immune cells. ‘Cold’ tumours are those without such infiltration or other features of an inflammatory response. Immune exclusion tumours are also characterised when immune cells are found only at the periphery of the lesion or in the stromal tissue of the tumour. Patients with resectable NSCLC are also being enrolled in a study of relatlimab, another anti-LAG-3 antibody (BMS-986016), as a neoadjuvant therapy [66]. Furthermore, an approach based on combining different inhibitors has already been proven effective in melanoma, another type of cancer. A combination of relatlimab and nivolumab (anti-PD-1 mAb) in phase 3 study RELATIVITY-047 for previously untreated metastatic or unresectable melanoma provided a longer PFS than nivolumab in monotherapy (10.1 months versus 4.6 months, *p* = 0.006) [67]. The promising results of research on melanoma offer great hope for the use of relatlimab and nivolumab combinations in the immunotherapy of other neoplastic diseases. Certainly, this requires its own prospective clinical study.

## 3. Is there Time to Stimulate Positive Immune Checkpoints in Cancer Immunotherapy?

Immunotherapy aimed at blocking negative immune checkpoints has become the standard of care for many cancers. The suppression of anti-tumour immune cell activity in the tumour microenvironment is also well described in the literature. It should be remembered, however, that the reactivation of exhausted lymphocytes in the tumour microenvironment only by blocking negative checkpoints may not be sufficient to restore full anticancer activity of these cells. Therefore, it may be reasonable to act simultaneously on two checkpoints: negatively (i.e., unlocking the brake) and positively (i.e., stepping on the gas pedal). The characteristics of the most important positive checkpoints and the possibility of using their stimulation in immunotherapy are presented below.

### 3.1. OX40 Molecule with Stimulant Ability

OX40 (also described as CD134 or tumour necrosis factor receptor superfamily member 4 [TNFRSF4]) is a type I transmembrane glycoprotein expressed mainly by T cells (expression induced by TCR [T-cell receptor] stimulation) [68]. It is also found on NK cells, NKT cells, and neutrophils [68]. Its ligand, OX40L (CD252), is expressed by DCs, activated B cells, macrophages, and NK cells [68]. As for OX40 signalling, it has been postulated that it acts through nuclear factor kappa-light-chain-enhancer of activated B cells (NF-κB), mitogen-activated protein kinase (MAPK), nuclear factor of activated T cells (NFAT), and Bcl-2/XL-dependent anti-apoptotic pathways [69,70]. Unlike the molecules mentioned above, OX40 provides co-stimulatory signals to various subsets of T cells. Broader effects of OX40/OX40L interaction include enhancement of the Th1-mediated immune response; Th2 cell maintenance; inhibition of Treg-mediated suppression; and augmentation of cytotoxic T-cell survival, expansion, and function [71,72,73,74].

The role of OX40 in anti-tumour immunity has been demonstrated by activation of the cytotoxic response through cooperation of CD8^+^ T cells with CD4^+^ T cells and the enhancement of CD8-positive cell expansion by direct interaction [75]. Additionally, in vitro studies have shown that the OX40 molecule is involved in the recall response to tumour-associated antigens [76]. Another mechanism reverses the suppressive effects of Tregs via OX40 agonism [77,78]. OX40 engagement also showed anti-tumour effects in a murine cancer model [79]. Furthermore, OX40 was detected on TILs in NSCLC tissue, and its ligand was found on cancer cells [80].

Preliminary studies have been conducted using agonistic agents against OX40 to increase costimulation and therefore enhance T-cell function in cancer. A phase 1 study of 9B12, a murine agonistic anti-OX40 mAb, examined antigen-specific immune responses to a variety of immunogens as a secondary outcome measure in patients with metastatic solid malignancies. It was observed that this agent increased the proliferation of peripheral blood CD4^+^ FoxP3-negative and CD8-positive T lymphocytes, as well as enhanced humoural and cellular immunity [81]. Moreover, tumour regression of at least one metastatic lesion in 12 of 30 patients was observed [81]. Some authors have argued that, based on preclinical data, therapy targeting both OX40 (as an agonistic agent) and PD-L1 (as an inhibitory agent) has synergistic effects; thus, a combination approach may be more efficient [82,83]. A preliminary report of a phase 1b dose escalation study of OX40 agonist MOXR0916 in combination with atezolizumab in patients with advanced solid tumours showed good tolerability, and an expansion phase was conducted on selected tumour types (Table 2) [84].

### 3.2. Dual Role of the CD137 Molecule in NSCLC Patients

CD137 (also known as 4-1BB and TNFRSF9) is another co-stimulatory and activation-inducible surface molecule found on a variety of immunological cells, such as CD4-positive and CD8-positive T cells and NK cells, Tregs, DCs (including follicular DCs), monocytes, neutrophils, and eosinophils [85,86]. Its ligand is expressed mostly by APCs, but CD137 and CD137L expression is not restricted only to immune cells [86,87,88]. Molecules involved in CD137 signalling include NF-κB, Jun amino-terminal kinases/stress-activated protein kinases (JNK/SAPK), and p38 MAPK [89]. 4-1BB promotes cytotoxic response by enhancing the proliferation and activation of CD8^+^ T cells [90]. CD137 signalling also augments the survival of these cells via Bcl-2 family members [91]. Research on CD137 function using agonistic antibodies showed that NK cell proliferation and IFN-γ production is promoted by CD137 stimulation, and that primed CD4^+^ T cells are activated through CD137 but with subsequent promotion of their apoptosis [92].

What distinguishes CD137/CD137L interaction is the fact that the ligand may act as a receptor and transmit reverse signalling to APCs, thereby enhancing their activity [93]. In monocytes, such signalling leads to their differentiation to DCs and augmentation of the Th1/Tc1-mediated immune response by DCs [93]. This, along with direct CD8^+^ cell reactivation (e.g., through PD-1 blockade), may be relevant in cancer, as cell-mediated responses are one of the basic anti-tumour activities. CD137L expression has been detected in various tumour cell lines, including lung cancer [94]. Furthermore, Qian et al. demonstrated the inhibition of the proliferation and induction of apoptosis in cancer cells in NSCLC by reverse signalling [95]. Zhu et al. determined that CD137^+^ T cells are tumour-reactive and able to inhibit tumour growth [96]. In a related vein, Guo et al. demonstrated an increase in the percentage of CD4^+^ and CD8^+^ T cells and a decrease in the percentage of Tregs at tumour sites in a murine model of ovarian cancer as an effect of combined TIM-3 blockade and CD137 activation [97]. Other preclinical studies showed anti-tumour activity of CD137-targeted treatment [98].

One agonistic anti-CD137 agent is urelumab. Results from integrated safety studies indicated immunological activity, but severe dose-dependent liver toxicities and hepatotoxicity-related deaths were also reported [99]. Utomilumab (PF-05082566) in combination with pembrolizumab (anti-PD-1) showed, in contrast to urelumab, good tolerability in the phase 1b study, and complete or partial responses were observed in 6/23 patients (26.1%) with advanced solid tumours [100]. In another study with utomilumab as monotherapy, preliminary anti-tumour activity was noted: the ORR was 3.8%, the median PFS was 1.7 months, and the median OS was 11.2 months in patients with solid tumours [101]. A summary of the anti-CD137 trials is presented in Table 2.

## 4. Beyond Well-Known Anti-PD-1 Antibodies

In lung cancer immunotherapy, two groups of ICIs are widely used: anti-PD-1 as well as anti-PD-L1 antibodies. Now, one of the most important questions oncologists are facing is the following: Do we have other therapeutic options? Cemiplimab, an antibody targeting the PD-1 molecule, was tested as a first-line monotherapy in a group of patients with advanced NSCLC according to PD-L1 tumour cell expression (in a multicentre, global, open-label phase 3 EMPOWER-Lung 1 study). In the group of patients with PD-L1 expression on at least 50% of tumour cells, a median OS was not reached (95% CI: 17.9–NR) within cemiplimab treatment, but it was 14.2 months (95 CI: 11.2–17.5) in patients receiving platinum-based chemotherapy. Significant improvements in median PFS were also observed, which were 8.2 months in the cemiplimab group versus 5.7 months in the chemotherapy group [102]. Notably, cemiplimab monotherapy is already indicated for use (in the United States and in the European Union) as a first-line therapy for adult patients with NSCLC and high PD-L1 expression (≥50% of tumour cells), but without *EGFR*, *ALK*, or *ROS1* aberrations. Moreover, cemiplimab could be used in locally advanced NSCLC patients who are not eligible for radical chemoradiotherapy or in metastatic NSCLC patients [103,104]. Cemiplimab is also approved for cutaneous squamous cell carcinoma and basal cell carcinoma [105,106].

Spartalizumab (PDR001) is also a novel anti-PD-1 mAb, which was investigated in a one to two phase study in various groups of patients (some of whom received immunotherapy in earlier lines of treatment). The recommended phase 2 doses were selected as 400 mg every 4 weeks or 300 mg every 3 weeks. The maximum tolerated dose was not reached. Adverse events included those typical of therapy with other PD-1 antibodies. The response rate reached 3.4%, and partial responses occurred in two patients: one with an atypical carcinoid tumour of the lung and one with anal cancer (Table 2) [107].

Another antibody that works against the PD-1 molecule, sintilimab, was the subject of a phase 3 trial. Advanced or metastatic squamous NSCLC patients were enrolled in this study. The toxicity and effectiveness of sintilimab with gemcitabine and platinum-based chemotherapy (GP) were compared to the corresponding effects with a placebo plus GP. An interim analysis showed benefits in PFS for the sintilimab plus GP group versus the placebo plus GP group (hazard ratio = 0.536, 95% CI: 0.422–0.681). Median OS was not reached in either group, but in the sintilimab plus GP group, it was significantly improved. The incidence of grade ≥ 3 adverse events was similar between both groups (Table 2) [108].

## 5. New Antibody Production Process: Dual-Affinity Re-Targeting (DART) Antibody Technology

To address the problems associated with conventional monoclonal antibodies, researchers recently managed to produce stable bispecific antibodies that demonstrate activity both in vitro and in vivo. A structure called dual-affinity re-targeting (DART) is a molecule composed of two pairs of a variable heavy chain domain (VH) and a variable light chain domain (VL) and a polypeptide linker; therefore, it is bispecific [109]. Some preclinical and clinical studies have shown promising results in indicating anti-tumour response.

One of the investigated drugs is bintrafusp alfa, a bifunctional fusion protein targeting TGF-β (tumour growth factor beta) and PD-L1. The role of the PD-L1 molecule is widely known. Considering the function of TGF-β, it may also play a role in regulating immunological processes in TME and inhibiting host tumour immune surveillance [110]. Moreover, it has been found to be overexpressed in various types of cancers [111]. The expansion cohort of a phase 1 trial in patients with NSCLC previously treated with platinum-based chemotherapy showed that bintrafusp alfa (M7824) was effective and tolerable as a second-line treatment. At a 1200 mg dose, which was recommended in phase 2, patients with PD-L1-positive (≥1% PD-L1^+^ tumour cells) and PD-L1-high (≥80% PD-L1^+^ tumour cells) cell counts had ORRs of 36.0% and 85.7%, respectively. Median PFS was 5.5 months (95% CI: 1.3–11.0) in patients with low PD-L1 expression (≥1%–<80% PD-L1^+^ tumour cells) and 15.2 months (95% CI: 1.3–NR) in patients with high expression [112]. Another randomised study of bintrafusp alfa versus pembrolizumab as a first-line treatment in patients with PD-L1 expressing advanced NSCLC is ongoing (Table 2) [113].

Combination therapy, where a PD-1/PD-L1 inhibitor is combined with a CTLA-4 inhibitor, has been proven to be more effective than monotherapies in some types of cancer, but an increase in the incidence of adverse events was observed [114]. In many clinical trials assessing the cellular composition of neoplastic tissue, an increased proportion of double positive cells (CTLA-4^+^/PD-1^+^) was observed in comparison to healthy tissues. Therefore, it seems reasonable to block both of these receptors simultaneously in tumour tissue, but to avoid systemic toxicity through Treg depletion in peripheral tissues, the function of these cells should be maintained. Berezhnoy et al. reported that bispecific DART protein binding PD-1 and CTLA-4 in a first-in-human study (still ongoing) showed that a combination blockade had an acceptable safety profile and satisfactory objective response rate in multiple tumour types [115].

DART technology is not the only method currently being explored to create bispecific drugs. An anti-PD-1/CTLA-4 monovalent, bispecific antibody (‘DuetMab’) is being tested in a trial in patients with advanced solid tumours, and some partial responses have already been observed (Table 2) [116]. Thus far, the simultaneous blockade of two targets appears to offer new opportunities in lung cancer treatment.

## 6. Summary

To date, among the various immunological treatment options for NSCLC, the administration of ICIs has been proven to be the most effective method for restoring the function of T cells at the tumour site. However, it is not completely free from limitations. Specifically, not all patients respond to the therapy, and resistance occurs in some cases. These difficulties are being addressed by researchers testing the potential of new drugs based on blocking or activating molecules other than PD-1 and PD-L1, as well as completely new methods of producing antibodies with dual specificity. The various receptors described above play a significant role in immunological processes occurring at the tumour site. Additionally, inhibitory molecules are often co-expressed by immune cells in the tumour microenvironment, which creates the possibility of targeting them. Numerous clinical trials have shown promising outcomes and acceptable safety profiles, either in monotherapy or in combination with other agents.

Naturally, more research is required to confirm the mostly promising results obtained in the trials mentioned above. First, it would be necessary to more precisely determine the prevalence of these molecules in the tumour microenvironment. The relationship between their expression and tumour diagnosis, treatment, and other factors should also be investigated, and it is expected that this area of research will certainly develop in the near future. Second, because the studies are mostly in the preliminary stages, there is little information on the adverse effects and the exact mechanisms of action of the new ICIs and checkpoint agonists. For instance, it would be interesting to analyse the populations of lymphocytes that are stimulated and whether they are different from those stimulated after currently used anti-PD-1/PD-L1 agents. As one of the mechanisms of resistance to immunotherapy is the expression of other inhibitory molecules, there should be studies conducted to specifically evaluate the efficacy of new antibodies in the first line of treatment for different groups of patients, but also in PD. Finally, some of the studies presented combined previously used antibodies with new ones, and this appears to be a forward-looking approach. However, we must consider whether double blockade in these circumstances would result in a higher rate of side effects, or whether appropriate dosing of each monoclonal antibody could minimise side effects while maintaining the efficacy of the therapy. The blockade of multiple immune checkpoints may be crucial in cancer immunotherapy, especially in overcoming resistance in further treatment lines or preventing their appearance in the first line of therapy. Nevertheless, it can clearly be seen that further advances in the field of lung cancer immunotherapy are achievable.

## Figures and Tables

**Table 1:** The short summary of three different types of resistance to immunotherapy [7].

Timing of Systemic Progression	Type of Resistance	Description
Early (<3 months)	Primary resistance	Cancer does not respond to an immunotherapy strategy
Intermediate (3 months–2 years)	Adaptive resistance	Most cancer cells are recognized by the immune system, but some cells are equipped with protective mechanisms
Late (>2 years)	Acquired resistance	Cancer initially responds to immunotherapy but after a period of time progression is observed

Based on the article: Sharma et al., 2017; modified by authors.

**Table 2:** Chosen clinical trials investigating antibodies targeting immune checkpoints in therapies of patients with solid tumours including NSCLC.

**Trial ID**	Target	Treatment Method	Line of Treatment	Cancer Type	Primary End Points	Phase
NCT02964013	TIGIT PD-1	vibostolimab + pembrolizumab	1st or 2nd	solid tumours (including NSCLC)	DLTs	1
NCT04738487	TIGIT PD-1	vibostolimab + pembrolizumab versus pembrolizumab	1st	PD-L1^+^ metastatic NSCLC	OS, PFS	3
NCT04725188	TIGIT PD-1	vibostolimab + pembrolizumab or vibostolimab + pembrolizumab + docetaxel versus docetaxel	2nd	metastatic NSCLC	PFS	2
NCT03563716	TIGIT PD-L1	tiragolumab + atezolizumab versus placebo + atezolizumab	1st	advanced PD-L1-selected NSCLC	ORR, PFS	2
NCT02608268	TIM-3, PD-1	sabatolimab (MBG453) alone or sabatolimab + spartalizumab (PDR001)	1st or subsequent	advanced solid tumours (including NSCLC)	DLTs, ORR and others	1/2
NCT02817633	TIM-3, LAG-3, PD-1	TSR-022 (anti-TIM-3), TSR-033 (anti-LAG-3), TSR-042 (anti-PD-1), nivolumab and chemotherapy in different combinations	1st, 2nd or 3rd	advanced solid tumours (including NSCLC)	DLTs, ORR and others	1
NCT02460224	LAG-3, PD-1	ieramilimab (LAG525) + spartalizumab (PDR001)	1st or subsequent	advanced solid tumours (including NSCLC)	DLTs, ORR	1/2
NCT01644968	OX40	9B12	failure of all standard therapeutic options	metastatic solid malignancies	DLTs	1
NCT02410512	OX40, PD-L1	MOXR0916 + atezolizumab	failure of all standard therapeutic options	advanced solid tumours (including NSCLC)	DLTs	1
NCT00309023 NCT00612664 NCT01471210 (integrated)	CD137	urelumab	2nd or subsequent	advanced solid tumours and lymphoma	AEs, DLTs	1 or 2
NCT02179918	CD137, PD-1	utomilumab (PF-05082566) + pembrolizumab	failure of all standard therapeutic options	advanced or metastatic solid tumours	AEs, DLTs	1
NCT01307267	CD137	utomilumab	1st or subsequent	advanced malignancies (including NSCLC)	DLTs	1
NCT03088540	PD-1	cemiplimab versus chemotherapy	1st	NSCLC	OS, PFS	3
NCT02404441	PD-1	spartalizumab	averagely 4th	solid tumours (including NSCLC)	DLTs, ORR	1/2
NCT03629925	PD-1	sintilimab + platinum compounds + gemcitabine (GP) versus placebo + GP	1st	squamous NSCLC	PFS	3
NCT02517398	TGF-β PD-L1	bintrafusp alfa (bispecific)	2nd	solid tumours (including NSCLC)	TEAEs, DLTs, BOR	1
NCT03631706	TGF-β PD-L1	bintrafusp alfa (bispecific) versus pembrolizumab	1st	metastatic NSCLC with high PD-L1 expression	PFS, OS	3
NCT03761017	PD-1 CTLA-4	MGD019 (bispecific)	averagely 4th	solid tumours (including squamous NSCLC)	TEAEs	1
NCT03530397	PD-1 CTLA-4	MEDI5752 (bispecific)	1st or subsequent	advanced solid tumours (including NSCLC)	TEAEs, DLTs and others	1

AEs—adverse events, BOR—best overall response, DLTs—dose limiting toxicities, ORR—overall response rate, OS—overall survival, PFS—progression-free survival, TEAEs—treatment emergent adverse events.

**Table 3:** TIM-3 ligands and their role in cancer.

Ligand	TIM-3 Ligand Expression in NSCLC	Effects of Interaction of TIM-3 Ligand with TIM-3 Molecule	References
Galectin-9	tumour cells and TILs	Apoptosis in TIM-3^+^CD8^+^ TILs	[37,38]
CEACAM1 (carcinoembryonic antigen cell adhesion molecule 1)	tumour tissue (IHC)	Tc exhaustion, cell-mediated cytotoxicity suppression	[31,39,40]
HMGB1 (high-mobility group box 1)	Primary EpCAM^+^ epithelial tumour cells	Suppression of innate immune responses through the recognition of nucleic acids by Toll-like receptors and cytosolic sensors in DCs	[32]
PtdSer (phosphatidylserine)	tumour cells and tumour vasculature	meaning in cancer not described (clearance of apoptotic cells)	[34,35,36]

## Data Availability

Not applicable.

## Abbreviations

ALK—anaplastic lymphoma kinase, APCs—antigen-presenting cells, BOR—best overall response, CTLA-4—cytotoxic T lymphocyte antigen 4, DART—dual-affinity re-targeting, DCs—dendritic cells, DLTs—dose limiting toxicities, DNAM-1—DNAX accessory molecule-1, DoR—duration of response, EGFR—epidermal growth factor receptor, ICIs—immune checkpoint inhibitors, irAEs—immune-related adverse events, ITIM—immunoreceptor tyrosine-based inhibitory motif, LAG-3—lymphocyte-activation gene 3, mAb—monoclonal antibody, MAPK—mitogen-activated protein kinase, NF-κB—nuclear factor kappa-light-chain-enhancer of activated B cells, NK—natural killer, NR—not reached, NSCLC—non-small-cell lung cancer, OR—objective response, ORR—overall response rate, OS—overall survival, PD—progressive disease, PD-1—programmed death 1, PD-L1—programmed death ligand 1, PFS—progression-free survival, PtdSer—phosphatidylserine, PVR—poliovirus receptor, RECIST—response evaluation criteria in solid tumours, ROS1—ROS proto-oncogene 1, SD—stable disease, Tc—T cytotoxic cell, TEAEs—treatment emergent adverse events, Th—T helper cell, TIGIT—T-cell immunoreceptor with Ig and ITIM domain, TIL—tumour-infiltrating lymphocyte, TIM-3—T-cell immunoglobulin mucin-3, TME—tumour microenvironment, TNFRSF—tumour necrosis factor receptor superfamily, TPS—tumour proportion score, Treg—T regulatory cell
